# Dr. Searcy Dead

**Published:** 1885-09-20

**Authors:** 


					﻿EDITORIALS AND MISCELLANEOUS.
Dr. Searcy Dead.—We are pained to learn of the recent death of one of
our esteemed subscribers, Dr. D. B. Searcy, of Bolingbroke, Ga. He was in
his seventy-seventh year, and died of heart disease.
				

